# The Linked CENTURY Study: linking three decades of clinical and public health data to examine disparities in childhood obesity

**DOI:** 10.1186/s12887-016-0567-0

**Published:** 2016-03-09

**Authors:** Summer Sherburne Hawkins, Matthew W. Gillman, Sheryl L. Rifas-Shiman, Ken P. Kleinman, Megan Mariotti, Elsie M. Taveras

**Affiliations:** Boston College, School of Social Work, McGuinn Hall, 140 Commonwealth Avenue, Chestnut Hill, MA USA; Obesity Prevention Program, Department of Population Medicine, Harvard Medical School and Harvard Pilgrim Health Care Institute, Boston, MA USA; Penn Center for Health Care Innovation, Philadelphia, PA USA; Division of General Academic Pediatrics, Department of Pediatrics, Massachusetts General Hospital for Children, Boston, MA USA; Department of Nutrition, Harvard T.H. Chan School of Public Health, Boston, MA USA

**Keywords:** Birth certificates, Electronic health records, Health status disparities, Medical record linkage, Pediatric obesity

## Abstract

**Background:**

Despite the need to identify the causes of disparities in childhood obesity, the existing epidemiologic studies of early life risk factors have several limitations. We report on the construction of the Linked CENTURY database, incorporating CENTURY (Collecting Electronic Nutrition Trajectory Data Using Records of Youth) Study data with birth certificates; and discuss the potential implications of combining clinical and public health data sources in examining the etiology of disparities in childhood obesity.

**Methods:**

We linked the existing CENTURY Study, a database of 269,959 singleton children from birth to age 18 years with measured heights and weights, with each child’s Massachusetts birth certificate, which captures information on their mothers’ pregnancy history and detailed socio-demographic information of both mothers and fathers.

**Results:**

Overall, 74.2 % were matched, resulting in 200,343 children in the Linked CENTURY Study with 1,580,597 well child visits. Among this cohort, 94.0 % (188,334) of children have some father information available on the birth certificate and 60.9 % (121,917) of children have at least one other sibling in the dataset.

Using maternal race/ethnicity from the birth certificate as an indicator of children’s race/ethnicity, 75.7 % of children were white, 11.6 % black, 4.6 % Hispanic, and 5.7 % Asian. Based on socio-demographic information from the birth certificate, 20.0 % of mothers were non-US born, 5.9 % smoked during pregnancy, 76.3 % initiated breastfeeding, and 11.0 % of mothers had their delivery paid for by public health insurance. Using clinical data from the CENTURY Study, 22.7 % of children had a weight-for-length ≥ 95^th^ percentile between 1 and 24 months and 12.0 % of children had a body mass index ≥ 95^th^ percentile at ages 5 and 17 years.

**Conclusions:**

By linking routinely-collected data sources, it is possible to address research questions that could not be answered with either source alone. Linkage between a clinical database and each child’s birth certificate has created a unique dataset with nearly complete racial/ethnic and socio-demographic information from both parents, which has the potential to examine the etiology of racial/ethnic and socioeconomic disparities in childhood obesity.

## Background

Despite recent evidence that childhood obesity in the US may have plateaued or even decreased [1, 2], progress has not been universal. From 2008 through 2011, the prevalence of obesity in low-income children age 2–4 years decreased in 19 of 43 states and territories, but remained high overall with a prevalence of 14 % [2]. According to nationally-representative data, obesity rates have also decreased among 2- to 5-year-olds, resulting in a prevalence of 8 % [1]. However, racial/ethnic disparities persist. In 2011–2012, 4 % of preschool-age white children were obese, compared to 11 % of black children, and 17 % of Hispanic children [1]. In contrast, obesity rates among older children have remained stable over the past decade at 18–21 % and ethnic minority children continue to be at higher risk [1]. In 2011–2012, 13 % of 6–11-year-old white children were obese, compared to 24 % of black children, and 26 % of Hispanic children [1]. Examining the causes of racial/ethnic and socioeconomic disparities in childhood obesity could help inform preventive interventions among those populations at highest risk.

Life course epidemiology proposes that factors during peri- and post-natal periods may influence the development of obesity from early life through adulthood [3, 4]. Observational studies have shown that maternal smoking during pregnancy [5–7], excessive gestational weight gain [8–10], gestational diabetes mellitus (GDM) [11], and accelerated infant weight gain [6, 12, 13] are associated with higher risk for childhood obesity. Some, but not all studies, also suggest that breastfeeding is protective [14–17]. More recently, cesarean delivery [18, 19] and antibiotic exposure in the first year of life [20, 21] have been associated with childhood obesity. At a more macro-level, aspects of the built and socioeconomic environment, such as access to food, opportunities for physical activity, and neighborhood deprivation [22–28], have been associated with childhood obesity and may explain racial/ethnic differences in obesity [29–31].

However, the existing epidemiologic studies of early life risk factors have several limitations. Foremost, the majority of research has been from observational studies of singletons, which are subject to confounding by genetic and shared environmental and familial factors. Given that randomized trials are often neither ethical nor feasible, alternative study methodologies, such as sibling pair designs [32], can reduce confounding and thus provide more valid inferences. Differences in outcomes between siblings can be compared when they have different exposures in utero or after birth, such as nicotine exposure if their mother smoked during one pregnancy but not the other. Since this methodology allows for partial control of the pre- and post-natal environment as well as shared genes [32, 33], it produces a less confounded estimate. If confounding is present, sibling-pair effect sizes would be smaller than those in an overall (between-family) analysis of the same data [33]. However, to date, there have been only a few sibling pair studies of any peri- or post-natal risk factors for childhood obesity [34–44]. Thus, whether many of the known risk factors are causally related to obesity remains unresolved.

In the US there are limited data sources that have information on peri- and post-natal risk factors, measured height and weight across childhood, racial/ethnic and socioeconomic diversity, and geocodes. Birth cohort studies [45, 46] have been invaluable resources because they collect detailed information on a range of exposure and outcome measures, but they often include a limited number of subjects and power to test interactions between race/ethnicity and measures of social class. Cohort studies also generally enroll only a single child from each family and, consequently, have limited sibling pairs.

Data linkage is a cost-effective approach to adding further value to routinely-collected data. State laws require that birth certificates be completed for all births and detailed information is collected on peri- and post-natal risk factors; however, health outcomes after discharge are not available. In contrast, clinical databases created from electronic health records contain child health outcomes, but information is often missing on socio-demographics and peri- or post-natal information. Linking these two sources of data can marry the advantages of each to overcome some of the noted limitations of previous study designs and help address the early origins of disparities in childhood obesity.

This paper first reports on the construction of the Linked CENTURY Study through data linkage between the CENTURY (Collecting Electronic Nutrition Trajectory Data Using e-Records of Youth) Study, a clinical database with measured height and weight data [47–49], with each child’s Massachusetts birth certificate; and second, discusses the potential clinical, epidemiologic, and public health implications of the Linked CENTURY Study in examining the etiology of disparities in childhood obesity.

## Methods

### CENTURY study

With funding from the Centers for Disease Control and Prevention in 1996, 2001, and 2008, we created the CENTURY Study, a database of children ages 0 to <18.0 years who were seen for a well child visit at any of the 14 health centers of Harvard Vanguard Medical Associates (HVMA) and other smaller health centers in eastern Massachusetts (currently Atrius Health) from 1980 through 2008. Originally a staff model health maintenance organization, HVMA evolved into a group practice in 1998. Its patients are predominantly employed and insured; children with Medicaid insurance were accepted from 1987 onwards. Since HVMA’s inception in 1969, it has used a completely electronic health record system for all medical encounters. To generate the CENTURY database, we obtained demographic and growth data from all well child visits from 1980 through 2008, for those children born from 1969 onwards. The definition of a well child visit was the use of an appropriate utilization code, the combination of measurement of weight and length or height, or administration of a routine immunization. The total sample size of the database is 306,147 children from birth to age 18 years with 2,110,014 well child visits from 1980 through 2008. Each child in the database was linked to his/her mother using insurance information and siblings were identified through a common family identifier. It is, therefore, possible that siblings may or may not be biological.

#### Measures from well child visits

##### Birth weight

Birth weight was extracted using both medical chart abstraction and text-search algorithms. Text-search algorithms use computational models that map clinical text to extract contextual use of words and phrases. Similar models have been used in electronic health records to identify adverse events of clinical care [50] and validate clinical diagnoses [51]. Birth weight is available in the CENTURY database for approximately 32 % of children.

##### Weights and lengths

Medical assistants measured length or height and weight according to the written protocol of the HVMA health centers. Weight was measured to the nearest 0.25 lb on a pediatric scale. Length in children < 24 months was measured recumbent. For children older than 36 months, height was generally measured standing. Medical assistants used a paper-and-pencil technique for children < 24 months rather than the recommended recumbent measuring board. In a measurement validation study conducted at one of the participating health centers, we found that this paper-and-pencil method systematically overestimated children’s length compared with the standard method. Thus, in all analyses of the CENTURY data, we correct recumbent length for children younger than 24 months using a regression correction factor from the validation study to adjust for this systematic overestimation [52].

We used measured height and weight to calculate age- and sex-specific weight-for-length (WFL) and body mass index (BMI) percentiles based on the Centers for Disease Control and Prevention (CDC) growth charts from 2000. The CDC defines obesity in children age 2–19 years as a BMI at or above the 95^th^ percentile for age and sex, with overweight between the 85^th^ and 95^th^ percentiles [53]. We used age- and sex-specific weight-for-length percentiles based on the 2000 CDC growth chart for children < 24.0 months [53].

##### Blood pressure

Medical assistants routinely take children’s blood pressure at well visits starting at age 3 according to the written protocol of the HVMA health centers. The protocol, which is based on recommendations from the American Heart Association [54], instructs patients to sit for five minutes before measuring blood pressure. It includes using a cuff that fits appropriately. Blood pressure is measured using automated or manual instruments, depending on what is available at each site.

We used clinical blood pressure readings to calculate age-, sex- and height-specific systolic blood pressure and diastolic blood pressure percentiles according to National Health Lung and Blood Institute guidelines [55].

##### Socio-demographic information

From the clinical record, we obtained the child’s gestational age, sex, age at the time of the visit, and type of medical insurance. Parental or clinician report of child’s race/ethnicity was recorded using the categories white, black, Hispanic, American Indian/Alaska Native, Asian, and other.

Due to the challenge of linking children from multiple birth pregnancies (i.e., twins, triplets) with their birth certificate, we retained 269,959 singleton children. Sample characteristics of the singleton children from the original CENTURY Study are shown in Table 1. All of the children had weight and height or length recorded at least once. However, information is missing on child’s race/ethnicity for 36 % of participants and medical insurance status for 66 % of participants.

**Table 1:** Sample socio-demographic characteristics, maternal health behaviors, and childhood obesity and blood pressure outcomes of the singleton children from the existing CENTURY study and Linked CENTURY study (linked with each child’s Massachusetts birth certificate), 1980–2008

	CENTURY Study		Linked CENTURY Study	
Characteristics	Total *N*	%	Total *N*	%
Any data at age ≤ 18 years	269959		200343	
Any data at age < 2 years	121389	45.0 %	104584	52.2 %
Any data at age 5 years	72195	26.7 %	57547	28.7 %
Any data at age 11 years	61270	22.7 %	44812	22.4 %
Any data at age 17 years	46559	17.2 %	31326	15.6 %
Race/ethnicity	Child’s race/ethnicity		Mother’s race/ethnicity	
White	121565	45.0 %	151643	75.7 %
Black	23906	8.9 %	23144	11.6 %
Hispanic	9018	3.3 %	9123	4.6 %
Asian	7941	2.9 %	11421	5.7 %
Other	11076	4.1 %	2396	1.2 %
Missing	96453	35.7 %	2616	1.3 %
Insurance	Medical insurance^d^		Delivery payment^a, e^	
Private	73412	27.2 %	136022	87.5 %
Medicaid	18215	6.7 %	17116	11.0 %
Other	-	-	1960	1.3 %
Missing	178332	66.1 %	398	0.3 %
Mother US born				
No			39972	20.0 %
Yes			158965	79.4 %
Missing			1406	0.7 %
Mother married at time of birth				
No			31232	15.6 %
Yes			169026	84.4 %
Missing			85	0.0 %
Mother smoked during pregnancy^b^				
No			109846	93.9 %
Yes			6852	5.9 %
Missing			264	0.2 %
Mother had gestational diabetes mellitus^c^				
No			80953	96.1 %
Yes			2884	3.4 %
Missing			413	0.5 %
Cesarean delivery^a^				
No			118439	76.2 %
Yes			36256	23.3 %
Missing			801	0.5 %
Breastfeeding initiation^a^				
No			36097	23.2 %
Yes			118638	76.3 %
Missing			761	0.5 %
Weight-for-length ≥ 95th percentile anytime between 1 and 24 months [69]	27331	22.5 %	23756	22.7 %
BMI ≥ 95th percentile [53]				
Age 5 years	8566	11.9 %	6934	12.0 %
Age 11 years	9619	15.7 %	7184	16.0 %
Age 17 years	5566	12.0 %	3758	12.0 %
Systolic blood pressure z-score [55]	*N*	Mean (SD)	*N*	Mean (SD)
Age 5 years	66391	−0.19 (0.81)	52997	−0.20 (0.81)
Age 11 years	57935	0.02 (0.89)	42391	0.02 (0.89)
Age 17 years	44652	−0.11 (0.97)	30078	−0.11 (0.97)
Diastolic blood pressure z-score [55]	*N*	Mean (SD)	*N*	Mean (SD)
Age 5 years	66391	0.08 (0.71)	52997	0.08 (0.71)
Age 11 years	57935	0.17 (0.74)	42391	0.17 (0.73)
Age 17 years	44652	0.11 (0.75)	30078	0.10 (0.74)

^a^From 1987

^b^From 1992

^c^From 1996

^d^Type of medical insurance at most recent visit recorded in clinical database

^e^Medical insurance status for the delivery recorded on the birth certificate

### Massachusetts Department of Public Health (MDPH) birth certificate data

Information on all live births in Massachusetts is stored in the Registry of Vital Records and Statistics at MDPH. The Massachusetts Standard Certificate of Live Birth, referred to as the ‘birth certificate’, consists of a Parent Worksheet and a Hospital Worksheet. The parent(s) completes the Parent Worksheet, which contains legal and socio-demographic information on the child’s mother and father. While the birth certificate does not confirm that the father is biological, it states that the information provided is about the child’s father regardless of whether the father will appear on the child’s legal birth record. A designated hospital representative (e.g., doctor, nurse, or hospital birth registrar) completes the Hospital Worksheet, which contains information on prenatal care, labor and delivery, neonatal conditions and procedures, and discharge.

#### Birth certificate measures

##### Pregnancy/infant measures

The birth certificate contains information on infant’s sex, birth weight, plurality, gestational age based on the last menstrual period and clinical estimates, mode of delivery, and parity.

##### Maternal health behaviors

Mothers self-report the average number of cigarettes they smoked daily before and, separately, during pregnancy. The hospital records the mother’s total weight gain/loss, whether the mother had GDM, whether the mother had hypertension, whether the mother was breastfeeding at the time the birth certificate was completed (referred to as breastfeeding initiation), and month prenatal care began and the number of prenatal care visits.

##### Socio-demographic information

Mothers and fathers each report their race (white, Black, Asian/Pacific Islander, American Indian, and other), age, place of birth, education, language preference, and marital status (mothers only). The birth certificate in Massachusetts also collects information on each parent’s ancestry or ethnic heritage (referred to as ethnicity) from 39 items, including several write-in options [56]. The hospital records the mothers’ medical insurance status for the delivery.

##### Geographic information

Mothers report the city and zip code of their residential mailing address on the birth certificate and the Registry reports the census tract. We have the ability to link each child’s census tract with area-level measures of socioeconomic circumstances through the census and the built environment. Information from commercial databases on locations of parks, fast food restaurants and supermarkets can provide indicators of children’s physical activity and food environments.

The birth certificate has undergone multiple revisions since its inception. While a majority of the variables from the birth certificate are available from 1969 onwards, when birth certificate data are first available from MDPH, birth certificates have collected increasingly more information over time. Data for the pregnancy/infant measures as well as maternal race, education, and marital status are available over the entire study period. Data for ethnicity and other socio-demographic characteristics are available primarily from 1987. Similarly, breastfeeding initiation, total weight gain/loss, and pregnancy-related hypertension were included in the birth certificate from 1987. Maternal smoking during pregnancy was collected from 1992 and GDM from 1996.

IRB approval for the Linked CENTURY Study was obtained from Boston College, Harvard Pilgrim Health Care (HPHC), MDPH, and Massachusetts General Hospital. Only approved study personnel at HPHC and MDPH had access to names and dates of birth for data linkage purposes and researchers had access only to a de-identified dataset.

#### Linkage procedure

**Fig. 1:**
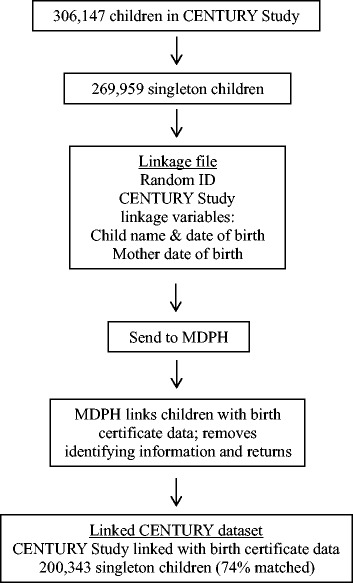
Flow diagram for linking the CENTURY Study data with each child’s Massachusetts birth certificate

In collaboration with MDPH, we developed a process for transferring the data between institutions (Fig. 1) and linking the datasets (Table 2). The Research Support Data Center at HPHC created a dataset that contained a random ID for each CENTURY Study child, child’s name and date of birth, mother’s date of birth, and all study variables. The Research Support Data Center sent the dataset to MDPH who linked each child with their birth certificate based on a linkage algorithm comparing the child’s name and date of birth and the mother’s date of birth. Table 2 presents the matching phase linkage algorithm and resulting number of matches for the six permutations of the algorithm. The majority of matches occurred only using the child information: 45.2 % of matches were based on the child’s first and last name and date of birth, while a further 33.3 % of matches were based on the child’s first, middle initial, and last name and date of birth. MDPH then removed identifying information and returned the dataset to our study team.

**Table 2:** Success rate of linkage algorithm by type of match (*N* = 200,343)

Matching phase	Type of match	Number linked	%
1	Child’s first, middle, and last name & dob and mother’s dob	5282	2.6 %
2	Child’s first, middle initial, and last name & dob and mother’s dob	32094	16.0 %
3	Child’s first, middle, and last name & dob	3035	1.5 %
4	Child’s first, middle initial, and last name & dob	66730	33.3 %
5	Child’s first and last name & dob	90506	45.2 %
6	Child’s first 3 letters of first name and last name & dob	2696	1.3 %

## Results

**Fig. 2:**
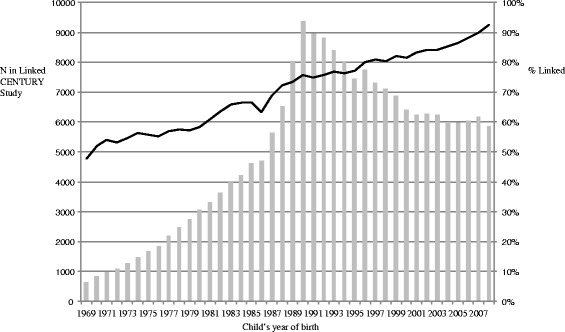
Number of participants in Linked CENTURY Study (*left axis - bars*) and % linked (*right axis - line*) by child’s year of birth (*N* = 200,343)

Overall, 74.2 % of the 269,959 singleton children were matched, resulting in 200,343 children in the Linked CENTURY Study with 1,580,597 well child visits. On average, each child had 7.9 visits (SD 6.6), range 1–93. The proportion of children who were linked to their birth certificate was higher in recent years from 47.8 % in 1969 to 92.4 % in 2008 (Fig. 2). As a result, 77.6 % of the children in the dataset were born from 1987 onwards. Differences in the proportion of children linked may be a result of when changes in the birth certificate were introduced (i.e., new items were added in 1987) (personal communication with Kevin Foster, October 14, 2014). Within this cohort, 60.9 % (121,917) children have at least one other sibling in the dataset.

Sample socio-demographic characteristics, maternal health behaviors, and childhood obesity and blood pressure outcomes of Linked CENTURY Study children are shown in Table 1. Approximately half of the sample had height/weight data available between 1 and < 24.0 months, one-third at age 5, and one-fifth at age 11 years. There is a 91 % agreement between maternal race/ethnicity from the birth certificate and children’s race/ethnicity from the existing CENTURY Study. Using maternal race/ethnicity as an indicator of children’s race/ethnicity, 75.7 % of children were white, 11.6 % black, 4.6 % Hispanic, 5.7 % Asian, and only 1.3 % had missing data. Using medical insurance status from the birth certificate as an indicator of socioeconomic circumstances, 11.0 % of mothers had their delivery paid for by public health insurance and 0.3 % had missing information.

Based on socio-demographic information from the birth certificate, 20.0 % of mothers were non-US born, 15.6 % were not married at the time of birth, 5.9 % smoked during pregnancy and 76.3 % initiated breastfeeding. Using clinical data from the CENTURY Study, 22.7 % of children had a weight-for-length ≥ 95^th^ percentile between 1 and 24 months and 12.0 % had a BMI ≥ 95^th^ percentile at ages 5 and 17 years.

Using clinical data from the CENTURY Study, 92 % of children with a visit at age 5 years had blood pressure measurement, 95 % at 11 years and 96 % at 17 years. At ages 5, 11, and 17 years, mean (SD) systolic blood pressure mm Hg values were 93.0 (8.7), 105.8 (9.8), and 114.5 (10.6) and mean (SD) diastolic blood pressure mm Hg values were 55.8 (8.0), 64.2 (8.3) and 68.9 (8.3). Blood pressure z-scores are provided in Table 1.

We compared children who were successfully linked with their birth certificate and those who were not linked (Table 3). Overall, differences by sex were minimal. Children not linked were more likely to be born in the 1970s and 1980s, from an ethnic minority group, or have missing race/ethnicity or medical insurance information.

**Table 3:** Socio-demographic characteristics from the CENTURY Study of children included in the Linked CENTURY Study and those who were not linked

	Linked CENTURY Study	Not linked
	(*N* = 200343)	(*N* = 69616)
Characteristics	%	%
Child’s year of birth (decade)		
1970s	8.6	19.8
1980s	23.9	34.0
1990s	40.0	33.2
2000s	27.6	13.0
Child’s sex		
Male	50.3	51.0
Female	49.7	49.1
Child’s race/ethnicity		
White	49.1	33.3
Black	7.7	12.1
Hispanic	2.7	5.3
Asian	2.7	3.8
Other	3.5	5.8
Missing	34.3	39.8
Medical insurance^a^		
Private	29.5	20.6
Medicaid	6.8	6.5
Missing	63.7	72.9

^a^Type of medical insurance at most recent visit recorded in clinical database

A feature of the Linked CENTURY Study is that 94.0 % (188,334) of children have some father information available. Table 4 compares the socio-demographic information from the birth certificate between mothers and fathers. Fathers were slightly older at the time of birth (mean 32 versus 30 years) and more likely to have 16+ years of education than mothers (18.2 % versus 14.0 %); however, there were few differences by race/ethnicity or nativity.

**Table 4:** Maternal and paternal socio-demographic information from the birth certificate

	Maternal (*N* = 200,343)		Paternal (*N* = 188,334)	
Characteristic	*N*	Mean (SD) or %	*N*	Mean (SD) or %
Age, years	199986	30 (6)	175425	32 (6)
Race/ethnicity				
White	151643	76.7 %	146572	79.0 %
Black	23144	11.7 %	18487	10.0 %
Hispanic	9123	4.6 %	7992	4.3 %
Asian	11421	5.8 %	10105	5.5 %
Other	2396	1.2 %	2334	1.3 %
Education				
<12 years	2433	1.2 %	2070	1.1 %
12 years	67141	34.0 %	64515	35.1 %
13–15 years	100254	50.8 %	83868	45.6 %
16+ years	27672	14.0 %	33489	18.2 %
US born				
No	39972	20.1 %	39671	21.5 %
Yes	158965	79.9 %	144668	78.5 %

Although the Linked CENTURY Study included children from eastern Massachusetts only, we compared selected maternal socio-demographic characteristics between singleton children born from 2004 to 2008 and all Massachusetts births in 2008 [57] (Table 5). Both datasets had similar proportions of mothers who were Black, US born, had GDM, and a cesarean delivery. The Linked CENTURY Study had more white (73.2 %) and Asian mothers (11.4 %) and fewer Hispanic mothers (4.9 %) than all Massachusetts births (67.2 %, 7.7 %, 14.2 %, respectively). While the Linked CENTURY Study had fewer mothers not married at the time of birth (17.7 % versus 24.0 %), mothers were more likely to have initiated breastfeeding (86.6 % versus 80.8 %) than all Massachusetts births.

**Table 5:** Comparison of selected maternal socio-demographic characteristics and health behaviors in the singleton children from the Linked CENTURY Study, births from 2004 to 2008, and all Massachusetts births in 2008 [57]

	Linked CENTURY Study		Massachusetts births	
	2004–2008 (*N* = 30,016)		2008 (*N* = 76,969)	
Maternal characteristics	Total *N*	%	Total *N*	%
Race/ethnicity				
White	21947	73.2	51,760	67.2
Black	2610	8.7	6,652	8.6
Hispanic	1471	4.9	10,895	14.2
Asian	3413	11.4	5,958	7.7
Other	544	1.8	-	-
Not US born	7935	26.4	21,299	27.7
Not married at time of birth	5323	17.7	26,146	34.0
Had gestational diabetes mellitus	1240	4.1	3,086	4.0
Cesarean delivery	9700	32.4	26,240	34.3
Initiated breastfeeding	25959	86.6	61,033	80.8

## Discussion

By linking routinely-collected data sources, we can address research questions that could not be answered with either source alone. Linkage of the existing CENTURY Study, a clinical database, with each child’s birth certificate, a public health data source, has created a dataset with the potential to examine the etiology of racial/ethnic and socioeconomic disparities in childhood obesity. The Linked CENTURY Study is a cohort of 200,343 children who can be followed through age 18 years. Future data extractions can update the dataset with newer cohorts of children as well as extend the longitudinal nature of the dataset for the existing children from 2008 through present.

There are many advantages to the type of data linkage we report. Harvesting data from electronic health records allowed us to generate a large, diverse cohort of children, which has the potential to be updated with more recent height and weight data or other items through future data extractions. Linking databases is a cost-effective study design for examining research questions using a life course perspective. Although the process of working with MDPH and HVMA was time consuming for the study team and personnel time should not be under-estimated, the physical cost of linking the data sources was less than $1,000. This price is substantially less than the cost of developing a cohort with primary data collection and long-term follow up. Linking databases has enabled us to fill in information that was missing in one source, but not the other. We can also conduct validation studies of an item from one source when the other can serve as a gold standard. In addition, most research on childhood obesity focuses on maternal or household indicators of socioeconomic status [1]. However, through the birth certificate data, we have the ability to look at the influence of both parents. The Linked CENTURY Study has socio-demographic information on the fathers of almost 190,000 children. Finally, little is known about the role of the neighborhood in explaining disparities in childhood obesity because geographical data are often not collected or available. Census tract information from the birth certificate will allow us to link to additional sources and examine the role of both area-level socioeconomic indicators and measures of the built environment on childhood obesity. While geographic information is currently only available from the child’s place of birth, the study team is exploring data extraction of the current residential address.

Most epidemiological studies examining risk factors for childhood obesity have been observational and, consequently, evidence has been based on associations. There are nearly 122,000 siblings in the Linked CENTURY. Sibling pair methodologies will allow us to reduce confounding by better controlling for genetic and shared environmental and familial factors [32]. Currently, only a limited number of sibling pair studies have examined early life risk factors, including smoking during pregnancy [35, 36], GDM [40, 44], gestational weight gain [41, 42], and breastfeeding [34, 37–39, 43]. We are not aware of sibling pair studies on accelerated infant weight gain and none of the more recent risk factors such as cesarean delivery or antibiotic use. With geographical data, it is also possible to explore differences in neighborhood effects between siblings who moved residence throughout childhood. Alternative methodologies to observational studies will produce less biased estimates and, ultimately, insights into areas for prevention. The study team has presented on several analyses using siblings in the Linked CENTURY Study to compare childhood obesity outcomes within families [58–60].

There are also a number of limitations that should be addressed. Linking datasets across institutions can be very time-intensive. In addition to the time that is required to apply for IRB approval from each institution, data confidentiality agreements and developing linkage algorithms can take many years. In addition, some institutions may have never been in contact previously and it can take time to develop these relationships.

Since some routinely-collected data are not objectively measured, there may be potential misclassification. Child race/ethnicity in the CENTURY Study was collected by either the parent or clinician, but it is not possible to determine who reported it. Some of the health-related items on the birth certificate are reported by the parent(s) or a hospital representative. For example, a mother reports on the average number of cigarettes she smoked during pregnancy on an average day. Maternal smoking during pregnancy is under-reported on the birth certificate compared to information on smoking collected on confidential surveys completed postpartum [61]. A hospital representative records yes or no in response to ‘is mother breastfeeding’, which serves as an indicator of breastfeeding initiation. In this case, a study in Massachusetts demonstrated a high level of agreement between the birth certificate and hospital infant feeding records [62]. The item of maternal total weight gained/lost is reported by the hospital at the time of delivery, but not necessarily based on measured weight and information on pre-pregnancy weight is not recorded. Validation studies have found misreporting of weight gain among women with a high body mass index or at the extremes of gestational weight gain [63, 64], posing some challenges for examining gestational weight gain using birth certificate data.

Attrition and selection bias in linked datasets are threats to internal validity similar to those in prospective cohort studies. There are two sources of missing data in our study. First, if children leave the clinical practice, then they will no longer be in our dataset. Second, some children have simply not aged into a category, i.e., children born after 1997 had not yet reached age 11. While 28.7 % of children have data at 5 years, only 22.4 % of children have data at 11 years. Extracting data from children’s electronic health records from 2009 onwards will increase the sample size at these later ages. A further limitation of clinical databases is that they often under-represent diverse populations who have less access to clinical care. HVMA accepted children with Medicaid insurance from 1987 onwards, suggesting that the database is less likely to be representative in prior years. However, using recent data, many of the maternal characteristics in the Linked CENTURY Study are similar to those for all births in Massachusetts (Table 5). Although the Linked CENTURY Study includes more mothers who were white and married at the time of birth, data specific to Eastern Massachusetts are not available.

Increasing the use of electronic health records to improve the coordination of care is an important feature of the Patient Protection and Affordable Care Act [65]. Internationally, data linkage is an active component of evaluating health system performance [66] and, ultimately, improving care and population health. Learning from new data linkage projects in the US [67–69] and more established ones in Europe [70–72] will provide further evidence on the potential for data linkages with electronic health records to address important public health problems like childhood obesity.

## Conclusions

Childhood obesity is prevalent, of consequence, has its origins in the earliest stages of life, and disproportionately affects children from racial/ethnic minority groups and from disadvantaged backgrounds. The Linked CENTURY Study, created by incorporating clinical data with birth certificates, is a unique dataset with nearly complete racial/ethnic and socio-demographic information from both parents. Thus, the Linked CENTURY Study has the potential to examine the etiology of racial/ethnic and socioeconomic disparities in childhood obesity.

## Abbreviations

BMI: Body mass index
CDC: Centers for Disease Control and Prevention
dob: date of birth
GDM: Gestational diabetes mellitus
HPHC: Harvard Pilgrim Health Care
HVMA: Harvard Vanguard Medical Associates
MDPH: Massachusetts Department of Public Health
WFL: Weight-for-length
